# The p38 MAPK Signaling Activation in Colorectal Cancer upon Therapeutic Treatments

**DOI:** 10.3390/ijms21082773

**Published:** 2020-04-16

**Authors:** Angelina Pranteda, Valentina Piastra, Lorenzo Stramucci, Deborah Fratantonio, Gianluca Bossi

**Affiliations:** 1 Oncogenomic and Epigenetic Unit, Department of Diagnostic Research and Technological Innovation, IRCCS—Regina Elena National Cancer Institute, Via Elio Chianesi 53, 00144 Rome, Italy; angelina.pranteda@ifo.gov.it (A.P.); piastra.valentina@gmail.com (V.P.); lorenzo.stramucci@gmail.com (L.S.); 2 Department of Biosciences, Biotechnologies and Biopharmaceutics, University of Bari Aldo Moro, Via Orabona 4, 70125 Bari, Italy; deborah.fratantonio@uniba.it

**Keywords:** p38 MAPK, colorectal cancer, therapeutic treatments, 5-fluorouracil, oxaliplatin, irinotecan, radiotherapy, target therapy

## Abstract

Pharmacological treatment of colorectal carcinoma currently proceeds through the administration of a combination of different chemotherapeutic agents. In the case of rectal carcinoma, radiation therapy also represents a therapeutic strategy. In an attempt at translating much-needed new targeted therapy to the clinics, p38 mitogen activated protein kinase (MAPK) inhibitors have been tested in clinical trials involving colorectal carcinoma patients, especially in combination with chemotherapy; however, despite the high expectations raised by a clear involvement of the p38 MAPK pathway in the response to therapeutic treatments, poor results have been obtained so far. In this work, we review recent insights into the exact role of the p38 MAPK pathway in response to currently available therapies for colorectal carcinoma, depicting an intricate scenario in which the p38 MAPK node presents many opportunities, as well as many challenges, for its perspective exploitation for clinical purposes.

## 1. Introduction

Colorectal cancer (CRC) is the third most common cancer, the fourth most common cause of cancer death, and the second most common cancer in terms of the number of individuals living with cancer five years after diagnosis worldwide [1]. CRC is a complex disease with a variable clinical course and with important divergences in the response to treatment, even in tumors with similar histopathological features. A recurrence rate of 33% was reported in CRC patients with stages II and III and 73% in metastatic stage IV CRC patients undergoing potentially curative resections [2]. Surgery remains the only curative option for patients with localized and locoregional CRC as well as for those with resectable distant metastases. Currently, 5-fluorouracil (5-FU), together with oxaliplatin or irinotecan, represents the cornerstone for CRC treatments [3]. Despite recent advances, while adjuvant therapy is effective at the early stages of the disease, resistance is conventionally observed in advanced stages, where treatment becomes ineffective. Therefore, a deeper understanding of the molecular mechanisms involved in CRC therapeutic responses would allow the identification of more efficient therapeutic strategies in CRC.

The stress-activated p38 mitogen activated protein kinase (MAPK) is one of the members of the superfamily of MAPKs [4]. Four p38 MAPK isoforms have been identified, namely α, β, γ, and δ, with α and β being the most abundant in a variety of tissues. The activation of this pathway usually proceeds through a conventional phosphorylation cascade, in which a MAPKKK (e.g., ASK1, TPL2, MEKK3) phosphorylates and activates the two main MAPKKs (MKK3 and 6) which, in turn, mediate the activation of the different p38 MAPK isoforms [5,6]. The p38 MAPK pathway activation is triggered by a variety of stimuli, including UV damage, oxidative stress, and exposure to DNA damaging agents, as well as growth factors and cytokines [7,8]. Upon activation, p38 MAPK regulates a plethora of cellular processes ranging from apoptosis to cell division, cell invasion, and inflammatory response [6,9].

Therefore, the p38 MAPK signaling pathway has been proposed as a critical node in cancer and its therapy [10,11,12]. Indeed, p38 MAPK signaling was reported as a major determinant of therapeutic efficacy of 5-FU, cisplatin, ara-c, and radiotherapy [13], and as major mediator in the resistance to several antitumor agents such as cisplatin, ara-c (cytosine arabinoside), or gemcitabine [14,15,16,17]. Coherently, p38 MAPK inhibition aimed at targeting the most abundant isoform, i.e., p38α MAPK, was explored in a variety of cancers. However, when translated into clinical practice, p38 MAPK manipulation did not meet the high expectations. We recently reported [18], at least in CRC, that 5-FU exposure induces p38δ MAPK isoform activation sustaining pro-survival signals; however, in parallel, others and us observed that the p38α MAPK isoform is also activated by 5-FU, and that this mediates the anti-tumor effect [12,19]. This simultaneous triggering of both anti-tumor and pro-survival effects goes along well with the pleiotropic role that the p38 MAPK signaling also exerts in physiological conditions. This demands a deeper characterization of the diverse players involved in the cascade and of their fine network of interactions with the p38 MAPK node; such insights are indispensable to define and correctly predict the outcome of the p38 MAPK pathway manipulation, especially in combination with other therapeutic regimens.

Here, we provide an overview of the recent findings in the field of p38 MAPK signaling pathway in CRC, focusing on its role in the response to drugs currently used in clinical practice.

## 2. 5-Fluorouracil Effects on the p38 MAPK Signaling Pathway in CRC

5-FU has been the clinical practice for the management of CRC (and other solid malignancies) for decades, and is currently used in combination with other chemotherapeutic agents [20,21]. 5-FU is a pyrimidine analogue that exerts its antitumoral function via both inhibiting thymidylate synthase, a crucial enzyme in DNA replication, and being misincorporated during polynucleotide biosynthesis, resulting into DNA damage and, ultimately, apoptosis [22]. The effects of 5-FU on molecular stress pathways have long been investigated: indeed, reports are quite unanimous about p38 MAPK being phosphorylated and activated upon 5-FU exposure in several different cell-types [23,24]. However, when it comes to the final biological outcome of 5-FU-induced p38 MAPK activation, reports are contradictive, assessing roles in both wild type p53 (wt-p53) mediated activation and apoptosis [25,26] or mediating prosurvival signaling via activation of DNA repair or antiapoptotic proteins [27,28]. Overall, the cellular context and interaction with other signaling pathways skew p38 MAPK signaling [5]. Also, in the case of CRC, reports are very unanimous about p38 MAPK being activated as a result of 5-FU exposure [12,24,25,28]. Nevertheless, studies assessing the effects of pharmacological inhibition of the p38 MAPK pathway with pyridinyl imidazole inhibitor (SB203580) revealed a divergent role for p38 MAPK activation in CRC. While it was originally proposed that p38 MAPK inhibition could sensitize CRC cells to 5-FU induced killing by exploiting HCT-116 cells [25], the opposite effect was observed when using a wider panel of CRC cell lines [12]. Indeed, genetic depletion of the p38α MAPK isoform (main target of the SB203580) also exerted protective effects against 5-FU induced apoptosis, confirming that p38α MAPK isoform activation mediates pro-apoptotic signaling in response to this chemotherapeutic agent [12], and hence, arguing against the use of p38 MAPK inhibitors as perspective therapeutic agents to be exploited in combination with chemotherapy [23]. More recently, it was demonstrated that, in response to 5-FU exposure, CRC cells display activation of the p38δ MAPK isoform and that depletion of either p38δ MAPK or its upstream kinase MKK3 (MAP2K3) univocally exerts antitumor effects [18]. This observation highlights the importance of isoform-specific activation of the p38 MAPK in mediating different effects and, in fact, provides a possible explanation for the contradictive effects reported for p38 MAPK activation in response to 5-FU. Indeed, while on one hand p38α MAPK mediates pro-apoptotic cues, p38δ MAPK also mediates pro-survival signaling, and it was recently suggested that both pathways could be activated in response to 5-FU exposure [19]. Indeed, it appears that pharmacologic p38α MAPK isoform inhibition still exerts protective effects against 5-FU induced apoptosis even in cells defective in p38δ MAPK prosurvival signaling (MKK3 depleted). Overall insights suggest that, in addition to an MKK3/p38δ MAPK prosurvival signaling, p38α MAPK proapoptotic signaling is triggered by 5-FU and possibly mediated by a different upstream kinase (likely, MKK6) [19].

**Figure 1:**
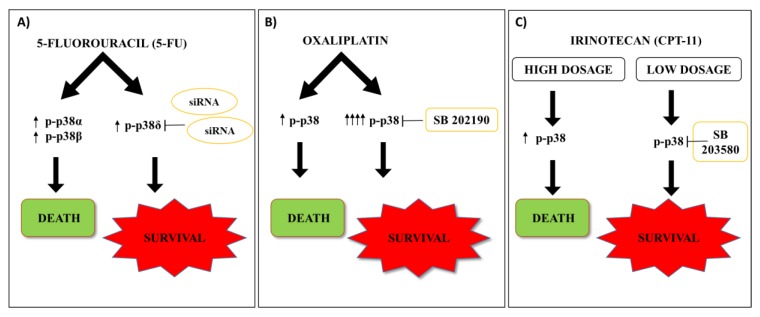
Chemotherapeutic agents in CRC. (**A**) 5-FU activates p38α and β isoforms leading to cell death, and the p38δ MAPK isoform, though MKK3 activation, leading to cell survival. Depletion of p38δ MAPK by RNA interference (siRNA) exerts antitumor effects. (**B**) Oxaliplatin induces apoptosis in CRC through p38 MAPK signaling pathway activation. The hyperactivation of p38 MAPK signaling supports oxaliplatin-resistance in CRC. Pharmacological p38 MAPK inhibition (SB 202190) re-sensitizes CRC cells to drug response. (**C**) Irinotecan (CPT-11) induces p38 MAPK activation dose dependently exerting different therapeutic outcomes in CRC: high dosage CPT-11 induces acute and short-lasting p38 MAPK activation leading to apoptosis; low dosage CPT-11 activates p38 MAPK in a delayed but sustained manner promoting cell-survival. Up arrows show levels of p38 activation via phosphorylation; down arrows show workflow of cell signaling; T bars represent inhibitory actions via siRNAs or chemicals.

Conclusively, while it is clear that p38 MAPK is activated upon 5-FU exposure, it appears that a deeper characterization of the molecular players involved in this complex signaling cascade will be needed to exactly define the role of p38 MAPK in the response to this specific agent, and that this will unlock new therapeutic targets for CRC [5] (Figure 1A).

## 3. Oxaliplatin Effects on the p38 MAPK Signaling Pathway in CRC

Oxaliplatin is a third-generation platinum-based drug that was considered among the most promising chemotherapy agents for CRC [29]. Oxaliplatin was the first drug from the diaminocyclo-hexane platinum family to be successfully developed in the clinic and is recommended as first-line chemotherapeutic agent. The most accredited mechanisms for oxaliplatin-induced anti-tumor effects include the ability to create cross-links and generate single and double-strand breaks in DNA, the ability to inhibit DNA and mRNA synthesis, and the ability to induce immunogenic cell death [30]. Nevertheless, oxaliplatin application in CRC as a monotherapy is restricted because of the occurrence of high toxicity caused by the high therapeutic doses and the development of drug resistance [31]. As a consequence, oxaliplatin is currently used only in combination with other therapeutics in CRC [29]. In vitro and in vivo evidence demonstrated that oxaliplatin induces apoptosis in CRC through the p38 MAPK signaling pathway activation [32]. Indeed, p38 MAPK activation was a required step in oxaliplatin induced anti-tumor effects in CRC lines, as demonstrated by the protective effect on oxaliplatin treated cells exerted by the p38 MAPK inhibitor SB202190 [33]. Such a finding was confirmed by a similar study exploiting the SB203580 p38 MAPK inhibitor [34,35]. Unexpectedly, the p38 MAPK-mediated anti-tumor effect did not appear to proceed through wt-p53 activation, as canonically reported [34] and, indeed, p38 MAPK inhibition protected from oxaliplatin exposure even in CRC cell lines lacking p53 functionality [35]. In partial agreement, oxaliplatin was reported to exert anti-tumor effects by inducing proteasomal degradation of Survivin as a result of p38 MAPK activation [32]. However, the same study also reported that p38 MAPK phosphorylation and activation depended on p53 activity [32], indicating that p53 status may, at least in part, skew the p38 MAPK-mediated oxaliplatin response. In sharp contrast, it was also reported that p38 MAPK is able to support resistance to oxaliplatin and that, indeed, oxaliplatin-resistant CRC cells both display hyperactivation of p38 MAPK in response to oxaliplatin exposure and re-sensitization to the drug upon p38 MAPK pharmacological inhibition [36]. These findings confirm that p38 MAPK modulation, even in the case of oxaliplatin, may mediate opposite effects, depending on the cell-specific context (Figure 1B).

## 4. Irinotecan Effects on the p38 MAPK Signaling Pathway in CRC

Irinotecan (CPT-11) is a derivative of camptothecin and it is used as a first-line chemotherapeutic drug to treat CRC [37]. It is converted in the organism to the pharmacologically active metabolite SN-38 by carboxylesterase. Like other camptothecin derivates drugs, CPT-11 acts as a topoisomerase I inhibitor, leading to lethal replication-mediated double-strand breaks [38]. Similarly to the other chemotherapeutics reviewed here, irinotecan exposure induces p38 MAPK activation in CRC: however, as it concerns the biological role of the p38 MAPK node, it still appears to be both dose and context-dependent. Indeed, studies on the SW620 CRC line revealed that, if on one hand, high CPT-11 dosage induces acute and short-lasting p38 MAPK activation leading to apoptosis mediated by mitochondria and caspases, low CPT-11 dosage also activates p38 MAPK in a delayed but sustained manner, with apoptosis occurring only in a fraction of cells and promoting overall cell-survival [39].

In agreement, CPT-11, when in co-treatments with proteasome inhibitor Carfilzomib (CFZ), revealed increased caspase 3 activity and CD95 expression along with marked increase in p38 MAPK activation in SW620 cells [40].

In line with these observations, a higher activation of p38 MAPK in SN-38 resistant clones derived from HCT-116 and SW480 CRC lines, as compared with the corresponding parental cells, was reported: moreover, p38 α and β isoforms were directly involved in the development of resistance to SN-38. Such an observation was mirrored in the fact that primary CRC samples from irinotecan-sensitive patients showed reduced levels of phosphorylated p38 MAPK with respect to CPT-11 non-responder patients, supporting the use of p38 MAPK pathway inhibition as a potential strategy to overcome resistance to CPT-11 based chemotherapies in CRC [41,42]. To further increase the complexity of the role of p38 MAPK signaling, studies reported the p38 MAPK activation as a required factor for the anti-metastatic activity of the phytochemical Thymoquinone, indicating that the plasticity of the p38 MAPK pathway is potentially involved even in this additional process [43] (Figure 1C).

## 5. Radiotherapy Effects on the p38 MAPK Signaling Pathway in CRC

**Figure 2:**
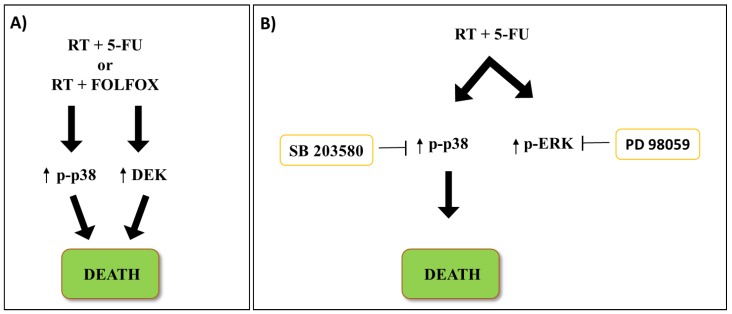
Radiotherapy (RT) in rectal cancer. (**A**) RT treatments revealed strong correlation of increased p38 MAPK and DEK levels with better therapeutic response when combined with 5-FU or FOLFOX in CRC patients. (**B**) RT combined with 5-FU treatment increases both p38 MAPK and ERK phosphorylated proteins levels. The p38 MAPK inhibition (SB203580) promotes cell survival, whereas ERK inhibition (PD98059) did not change treatment-related effects. Up arrows show levels of proteins activation; down arrows show workflow of cell signaling; T bars represent inhibitory actions via chemicals.

In the last decades, the interest towards radiotherapy (RT) as a strategy to treat CRC at different tumor stages from early to metastatic is constantly increasing [44]. Approximately one-third of CRC cases are of rectal origin [45]; while RT is rarely used in colon cancer, it is commonly adopted in rectal cancer [46], either administered alone or combined with chemotherapy [47]. A Swedish rectal cancer trial reported preoperative RT as a promising strategy because of its improved patient survival and reduced local failure rates [47]. The roles of p38 MAPK signaling in response to RT are not well defined and are only recently being addressed. Recent studies implicated p38 MAPK activation in the response to radiotherapy in CRC [48]; retrospective analysis on 74 rectal cancer patients revealed a strong correlation of p38 MAPK and DEK levels with better response to 5-FU or FOLFOX treatment, when combined with RT (Figure 2A). The expression of both proteins was clearly correlated with increased cell death, and thus better prognosis [48], suggesting the monitoring of both proteins as robust predictive model of therapeutic response. In line with that observation, Wang et al. [49] demonstrated that p38 MAPK, along with ATM/TP53 activation, is required for better outcomes in response to 5-FU combined with low or high dose irradiation. Interestingly, albeit treatments increased both the p38 MAPK and ERK phosphorylated and total proteins, p38 MAPK was the only factor directly involved in the response to RT, as assessed by rescue experiments exploiting MEK (PD98059) or p38 MAPK (SB203580) inhibitors [49]. Further studies reported 5-FU pre-treatment to radiosensitize CRC cells through p38 MAPK and wt-p53 activation, indicating a cooperativity of either chemotherapy potentiating RT or vice-versa [50] (Figure 2B). In fact, it was also demonstrated that p38 MAPK is involved in the regulation of G1/S checkpoint after γ radiation, although in a wt-p53 independent manner its effect was exerted through the phosphorylation of HuR (Hu antigen R), an RNA binding protein regulating the half-life of p21 RNA, causing p21 cytoplasmic accumulation and cell cycle arrest [51].

## 6. Role of the p38 MAPK Signaling Pathway in the Response to Other Therapeutic Strategies

CRC is a disease that does not present many biomarkers predicting a targeted response from biological inhibitors [52]. However, the effect of several candidate drugs, potential therapeutic targets, and natural compounds are tightly linked with the p38 MAPK pathway. Below we summarize most representative target therapy agents and their interactions with p38 MAPK signaling (Table 1).

**Figure 3:**
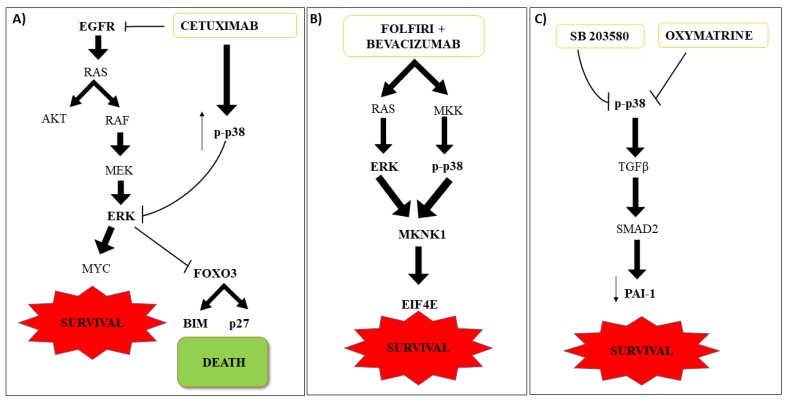
Target therapy in CRC. (**A**) Activated EGFR results in the phosphorylation of ERK and AKT signaling pathways, which in turn phosphorylate FOXO3a, priming it for nuclear translocation and degradation. In this condition, FOXO3a, BIM, and p27 are not transcribed, resulting in sustained cell proliferation and survival. p38 MAPK is able to inhibit ERK and to activate FOXO3, inducing apoptotic cell death by transcription of BIM and p27. Cetuximab therapy failure was associated with p38 MAPK inhibition. (**B**) FOLFIRI/Bevacizumab treatment increases MKNK1 protein level (MAPK-interacting kinase 1), the target of RAS/RAF/ERK and MKK/p38 MAPK signaling, regulating the activation of eIF4E. (**C**) Oxymatrine (OM) decreases PAI-1 and p38 MAPK protein levels. TGF-β1 signaling regulates PAI-1 through p38 MAPK signaling. The p38 MAPK inhibition down regulates pSmad2 and PAI-1, hampering invasion and cell migration. Up arrows show levels of p38 activation via phosphorylation; down arrows show workflow of cell signaling activation and inhibitory effects on PAI-1; T bars represent inhibitory actions via chemicals.

Cetuximab is an IgG1 chimeric monoclonal antibody against epidermal growth factor receptor (EGFR) with activity against CRC that express EGFR. The p38 MAPK signaling has been shown to be involved in therapy response in CRC KRASwt cells. EGFR activation results in the phosphorylation of ERK and AKT signaling pathways, which in turn phosphorylate FOXO3a, priming it for nuclear translocation and degradation. In this condition, the target genes of FOXO3a, BIM, and p27 are not transcribed, resulting in sustained cell proliferation and survival. Cetuximab inhibits ERK and activates FOXO3 through p38 MAPK activation, inducing apoptotic cell death by transcription of BIM and p27. Indeed, failure of Cetuximab therapy in some patients was associated with the inhibition of p38 MAPK via EGFR mediated activation of MKP-1 [53]. In line with these observations, high levels of MKP-1 were reported in metastatic CRC (mCRC) patients [54]. Overall insights suggest p38 MAPK as an important player in the treatments with EGFR inhibitors in CRC (Figure 3A).

Bevacizumab is a humanized monoclonal antibody that binds VEGF-A, preventing it from binding to its cognate receptor and activating a signaling cascade that leads to angiogenesis. Bevacizumab is used in mCRC in combination with chemotherapy to inhibit angiogenesis. The study of three cohorts of patients untreated or treated with combination FOLFIRI/Bevacizumab evidenced variations of MAPK-interacting kinase 1 (MKNK1) gene expression to correlate with progression-free survival (PFS). MKNK1 is target of both RAS/RAF/ERK and MKK/p38 MAPK pathways [55] and regulates the activation of eIF4E (eukaryotic translation initiation factor 4E), strongly upregulated in cancer patients and correlated with disease progression [56,57]. However, while MKNK1 could be a predictive biomarker of response in KRASwt mCRC patients treated with FOLFIRI/Bevacizumab [58], it is still unclear whether its activation proceeded through the RAS/RAF/ERK or the p38 MAPK pathway activation (Figure 3B).

Baicalein (5,6,7-trihydroxyflavone), extracted from the dry root of *Scutellaria baicalensis Georgi*, is recognized for a wide range of pharmacological functions and antitumor activity in several cancers [59,60,61]. The effect of Baicalein in CRC depends on the induction of activated caspase-3, caspase-9, and the JNK/ERK/p38 MAPK pathways leading to apoptosis in HCT116 CRC cells. However, the roles of MAPKs in mediating Baicalein-induced apoptosis remains to be elucidated [62].

Oxymatrine (OM), a drug extracted from the Chinese herb *Sophora flavescens ait*, has been reported to exert inhibitory effects on the growth of CRC via the p38 MAPK pathway. OM treatments decrease plasminogen activator inhibitor 1 (PAI-1) and p38 MAPK protein levels. PAI-1 is an important target of the TGF-β/Smad signaling pathway involved in invasion and cell migration through the degradation of the extracellular matrix. High levels of PAI-1 are related to a worse prognosis when reported in the plasma of metastatic rectal cancer patients [63] and some other tumors [64]. High p38 MAPK levels correlate positively with PAI-1 levels through UR and FHC groups. To verify the role of p38 MAPK signaling in response to OM treatments in CRC, RKO cells treated with SB203580 showed down regulation of pSmad2 and PAI-1, suggesting a role of p38 MAPK in Smad2 regulation and thus Smad2/3/4 complex formation [65]. In addition, p38 MAPK was reportedly involved in the regulation of PAI-1 protein expression mediated by the TGF-β1 signaling pathway [66] (Figure 3C).

In addition to the demonstrated active roles of p38 MAPK signaling in mediating drugs effects, its inhibition was reported upon treatment with different drugs like Rifaximin and Imperatonin in CRC. Rifaximin is an antibiotic used in therapy for its anti-inflammatory properties, and is an inhibitor of NF-κB and intestinal human pregnane X (PXR) receptors [67,68]. In Caco2 CRC cells, Rifaximin reduces PCNA protein levels and consequently decreases cell proliferation, also reducing mediators of cell migration and angiogenesis [69]. Rifaximin inhibits the activation of AKT, NF-κB, and p70S6K, leading to a reduction in HIF-1α levels and inhibition of the p38 MAPK signaling pathway [70]. Imperatorin, one of the major active coumarins found in the root of *Imperata cylindrica Beauv* with many pharmacological activities including anti-inflammatory, anti-coagulant, and anti-proliferative effects, was reported to greatly reduce HIF-1α levels by decreasing hypoxia in various types of tumors. This compound also was reported to exert effects on SAPK/JNK, p38 MAPK, mammalian phosphorylation target of rapamycin (mTOR), ribosomal protein S6 kinase (p70S6K), eIF4E binding protein-1 (4E-BP1), eukaryotic translation initiation factor 4E (eIF4E), and ERK1/2 [71] (Table 1).

## 7. Conclusions

The p38 MAPK node is clearly activated in response to all current therapeutic strategies for CRC; however, when it comes to the final biological outcome of such activation, the plasticity of the node itself poses several caveats to its manipulation for therapeutic purposes. Indeed, it appears that p38 MAPK isoform-specific activation, their acute or chronic modulation, as well as the specific context in which it occurs (e.g., sensitive or resistant clones) may result in distinct and contradictive effects. As it concerns p38 MAPK as a therapeutic target, the variability of response to treatment with p38 MAPK inhibitors in CRC is associated in literature with the pleiotropic nature of the different p38 MAPK isoforms and different levels of other proteins. Zhang et al. demonstrated that 20% of CRCs have a better response to p38 MAPK inhibitor treatments because they have low PP2AC levels while patients with higher expression levels of PP2AC are resistant to p38 MAPK inhibitors [72]. Such plasticity of a potential target for pharmacologic modulation requires a joint effort aimed both at estimating the overall effect of drug combinations in highly representative models and characterizing the fine-tuning of single components of the p38 MAPK node in response to each different cue in each different model. To try to depict a picture of the p38 MAPK functions in response to therapeutic strategies currently in CRC patients, we reviewed literature published in the last 10 years. The overall conclusion is still missing the univocal role of the p38 MAPK pathway/isoforms in the CRC therapeutic response. This is undoubtedly due to the pathway complexities, as above stated, but also to the experimental approaches adopted that are largely based on pharmacological p38 MAPK inhibition and primarily focused on p38 MAPK α and β isoforms, which are often poorer methods than the alternative experimental approaches, for instance genetic manipulation (RNA interference, RNAi), to further validate the achieved results. We recently demonstrated that p38δ MAPK, mainly activated by upstream MKK3 kinase in CRC, is further activated by 5-FU, thus hampering its efficacy. The p38δ MAPK inhibition by RNAi improves 5-FU response in CRC lines in vitro and in vivo [18]. Of interest with identical experimental CRC models, the pharmacologic p38α MAPK inhibition (SB203580) exerts protective effects against 5-FU induced apoptosis, suggesting that, in addition to an MKK3/p38δ MAPK pro-survival signaling, a p38α MAPK pro-apoptotic signaling is triggered by 5-FU and possibly mediated by a different upstream kinase (likely, MKK6) [19]. In this view, the in vitro and in vivo CRC models may fail at completely depicting the complex role exerted by the p38 MAPK in cancer progression and response to treatments, and clinical testing should be carefully evaluated accordingly in order to better tailor the exploitation of this central hub and maximize the clinical outcome for CRC patients. In conclusion, while there is a lack of expectations in p38 MAPK targeting in clinical trials, the p38 MAPK node presents still many opportunities, as well as many challenges, for its perspective exploitation for clinical purposes.

## Figures and Tables

**Table 1:** Drugs and relative molecular mechanisms.

Compound	Action	References
**Cetuximab**	Chimeric monoclonal antibody against epidermal growth factor receptor (EGFR). Its interaction prevents the binding to EGF inhibiting cell growth and survival.	[53,54]
**Bevacizumab**	Anti-VEGF recombinant monoclonal antibody. It inhibits VEGF receptors (VEGFR) preventing blood vessels proliferation.	[55,56,57,58]
**Baicalein (5,6,7-trihydroxyflavone)**	Flavone, type of flavonoid, originally isolated from the roots of *Scutellaria baicalensis*. Compound with anti-tumor activity, in several cancers, mainly due to its capacities to inhibit cyclins complexes and thus to regulate the cell cycle.	[59,60,61,62]
**Oxymatrine**	Potent monosomic alkaloid derived from the root of *Sophora flavescens Ait*. Compound with anti-inflammatory, anti-oxidative and hepatoprotective activities.	[63,66]
**Rifaximin**	Synthetic rifamycin derivative and anti-bacterial agent, used for the treatment of gastroenteritis by *Escherichia coli* infections. It may also be used in the treatment of hepatic encephalopathy.	[67,68,69,70]
**Imperatorin**	Tumor necrosis factor antagonist; furanocoumarin from West African medicinal plant *Clausena anisata*.	[71]

Drugs were selected for their therapeutic potential closely related to p38 MAPK pathway activation in regulatory mechanisms of survival, cell death, and malignancy in CRC. In order to enhance their therapeutic effects, these compounds were used in combination with chemotherapeutics.

## Abbreviations

5-FU	5-fluorouracil
4E-BP1	eIF4E binding protein-1
AKT ara-c ASK1	Protein kinase B Cytosine arabinoside Apoptosis signal-regulating kinase 1
ATM	Ataxia-telangiectasia mutated
CFZ	Carfilzomib
CPT-11	Irinotecan/Camptothecin-11
eIF4E	Eukaryotic translation initiation factor 4E
ERK1/2	Extracellular signal-regulated-1/2-ER2
FOLFOX	Folinic acid, 5-fluorouracil, Oxaliplatin
HIF-1α	Hypoxia-inducible factor 1-alpha
HuR	Hu antigen R
MAPKK/MKK	Mitogen-activated protein kinase kinase
MEKK3	Mitogen-activated protein kinase kinase kinase 3
MKP-1	Mitogen-activated protein kinase 1
MKNK1	MAPK-interacting kinase 1
OM	Oxymatrine
mTOR	Mammalian phosphorylation target of rapamycin
p70S6K	Ribosomal protein S6 kinase
PAI-1	Plasminogen activator inhibitor 1
PCNA	Proliferating Cell Nuclear Antigen
PFS	Progression-free survival
PP2AC	Serine/threonine-protein phosphatase 2A catalytic subunit alpha isoform
PXR	Pregnane X receptor
RT	Radiotherapy
SAPK/JNK	c-Jun N-terminal kinases
SN-38	7-etil-10-idrossi-camptotecina
SMAD	Small mother against decapentaplegic
TGFβ-1	Transforming growth factor beta 1
TP53	Tumor protein p53
TPL2	Tumor progression locus 2

## References

[1] Steelman L.S., Fitzgerald T., Lertpiriyapong K., Cocco L., Follo M.Y., Martelli A.M., Neri L.M., Marmiroli S., Libra M., Candido S. (2016). Critical Roles of EGFR Family Members in Breast Cancer and Breast Cancer Stem Cells: Targets for Therapy. Curr. Pharm. Des..

[2] Hall M.J., Morris A.M., Sun W. (2018). Precision Medicine Versus Population Medicine in Colon Cancer: From Prospects of Prevention, Adjuvant Chemotherapy, and Surveillance. Am. Soc. Clin. Oncol. Educ. Book.

[3] Raftery L., Sanoff H.K., Goldberg R. (2008). Colon cancer in older adults. Semin Oncol..

[4] Avruch J. (2007). MAP kinase pathways: The first twenty years. Biochim. Biophys. Acta.

[5] Stramucci L., Pranteda A., Bossi G. (2018). Insights of Crosstalk between p53 Protein and the MKK3/MKK6/p38 MAPK Signaling Pathway in Cancer. Cancers.

[6] Cuadrado A., Nebreda A.R. (2010). Mechanisms and functions of p38 MAPK signalling. Biochem. J..

[7] Keshet Y., Seger R. (2010). The MAP kinase signaling cascades: A system of hundreds of components regulates a diverse array of physiological functions. Methods Mol. Biol..

[8] Lee J.C., Laydon J.T., McDonnell P.C., Gallagher T.F., Kumar S., Green D., McNulty D., Blumenthal M.J., Heys J.R., Landvatter S.W. (1994). A protein kinase involved in the regulation of inflammatory cytokine biosynthesis. Nature.

[9] Cuenda A., Rousseau S. (2007). p38 MAP-kinases pathway regulation, function and role in human diseases. Biochim. Biophys. Acta.

[10] Olson J.M., Hallahan A.R. (2004). p38 MAP kinase: A convergence point in cancer therapy. Trends Mol. Med..

[11] Wagner E.F., Nebreda A.R. (2009). Signal integration by JNK and p38 MAPK pathways in cancer development. Nat. Rev. Cancer.

[12] De la Cruz-Morcillo M.A., Valero M.L., Callejas-Valera J.L., Arias-González L., Melgar-Rojas P., Galán-Moya E.M., García-Gil E., García-Cano J., Sánchez-Prieto R. (2012). P38MAPK is a major determinant of the balance between apoptosis and autophagy triggered by 5-fluorouracil: Implication in resistance. Oncogene.

[13] Hernández Losa J., Parada Cobo C., Guinea Viniegra J., Sánchez-Arevalo Lobo V.J., Ramón y Cajal S., Sánchez-Prieto R. (2003). Role of the p38 MAPK pathway in cisplatin-based therapy. Oncogene.

[14] Habiro A., Tanno S., Koizumi K., Izawa T., Nakano Y., Osanai M., Mizukami Y., Okumura T., Kohgo Y. (2004). Involvement of p38 mitogen-activated protein kinase in gemcitabine-induced apoptosis in human pancreatic cancer cells. Biochem. Biophys. Res. Commun..

[15] Koizumi K., Tanno S., Nakano Y., Habiro A., Izawa T., Mizukami Y., Okumura T., Kohgo Y. (2005). Activation of p38 mitogen-activated protein kinase is necessary for gemcitabine-induced cytotoxicity in human pancreatic cancer cells. Anticancer Res..

[16] Sánchez-Arévalo Lobo V.J., Aceves Luquero C.I., Alvarez-Vallina L., Tipping A.J., Viniegra J.G., Hernández Losa J., Parada Cobo C., Galán Moya E.M., Gayoso Cruz J., Melo J.V. (2005). Modulation of the p38 MAPK (mitogen-activated protein kinase) pathway through Bcr/Abl: Implications in the cellular response to Ara-C. Biochem. J..

[17] Galan-Moya E.M., Hernandez-Losa J., Aceves Luquero C.I., de la Cruz-Morcillo M.A., Ramírez-Castillejo C., Callejas-Valera J.L., Arriaga A., Aranburo A.F., Ramón y Cajal S., Silvio Gutkind J. (2008). c-Abl activates p38 MAPK independently of its tyrosine kinase activity: Implications in cisplatin-based therapy. Int. J. Cancer.

[18] Stramucci L., Pranteda A., Stravato A., Amoreo C.A., Pennetti A., Diodoro M.G., Bartolazzi A., Milella M., Bossi G. (2019). MKK3 sustains cell proliferation and survival through p38DELTA MAPK activation in colorectal cancer. Cell Death Dis..

[19] Brenner H., Kloor M., Pox C.P. (2014). Colorectal cancer. Lancet.

[20] Kuipers E.J., Grady W.M., Lieberman D., Seufferlein T., Sung J.J., Boelens P.G., van de Velde C.J., Watanabe T. (2015). Colorectal cancer. Nat. Rev. Dis. Primers..

[21] Zhang J., Yang P.L., Gray N.S. (2009). Targeting cancer with small molecule kinase inhibitors. Nat. Rev. Cancer.

[22] García-Cano J., Roche O., Cimas F.J., Pascual-Serra R., Ortega-Muelas M., Fernández-Aroca D.M., Sánchez-Prieto R. (2016). p38MAPK and Chemotherapy: We Always Need to Hear Both Sides of the Story. Front. Cell Dev. Biol..

[23] Grossi V., Peserico A., Tezil T., Simone C. (2014). p38α MAPK pathway: A key factor in colorectal cancer therapy and chemoresistance. World J. Gastroenterol..

[24] Can G., Akpinar B., Baran Y., Zhivotovsky B., Olsson M. (2013). 5-Fluorouracil signaling through a calcium-calmodulin-dependent pathway is required for p53 activation and apoptosis in colon carcinoma cells. Oncogene.

[25] Yang S.Y., Miah A., Sales K.M., Fuller B., Seifalian A.M., Winslet M. (2011). Inhibition of the p38 MAPK pathway sensitises human colon cancer cells to 5-fluorouracil treatment. Int. J. Oncol..

[26] Liu J.L., Huang W.S., Lee K.C., Tung S.Y., Chen C.N., Chang S.F. (2018). Effect of 5-fluorouracil on excision repair cross-complementing 1 expression and consequent cytotoxicity regulation in human gastric cancer cells. J. Cell Biochem..

[27] Bracht K., Nicholls A.M., Liu Y., Bodmer W.F. (2010). 5-Fluorouracil response in a large panel of colorectal cancer cell lines is associated with mismatch repair deficiency. Br. J. Cancer.

[28] Stramucci L., Bossi G. (2019). Approaching the challenges of MKK3/p38delta MAPK targeting for therapeutic purpose in colorectal cancer. J. Exp. Clin. Cancer Res..

[29] Wils J. (2007). Adjuvant treatment of colon cancer: Past, present and future. J. Chemother..

[30] Mehmood R.K., Parker J., Ahmed S., Qasem E., Mohammed A.A., Zeeshan M., Jehangir E. (2014). Review of Cisplatin and Oxaliplatin in Current Immunogenic and Monoclonal Antibodies Perspective. World J. Oncol..

[31] Alcindor T., Beauger N. (2011). Oxaliplatin: A review in the era of molecularly targeted therapy. Curr. Oncol..

[32] Dey H., Liu Z.R. (2012). Phosphorylation of p68 RNA helicase by p38 MAP kinase contributes to colon cancer cells apoptosis induced by oxaliplatin. BMC Cell Biol..

[33] Liu H.F., Hu H.C., Chao J.I. (2010). Oxaliplatin down-regulates survivin by p38 MAP kinase and proteasome in human colon cancer cells. Chem. Biol. Interact..

[34] Perfettini J.L., Castedo M., Nardacci R., Ciccosanti F., Boya P., Roumier T., Larochette N., Piacentini M., Kroemer G. (2005). Essential role of p53 phosphorylation by p38 MAPK in apoptosis induction by the HIV-1 envelope. J. Exp. Med..

[35] Rakitina T.V., Vasilevskaya I.A., O’Dwyer P.J. (2003). Additive interaction of oxaliplatin and 17-allylamino-17-demethoxygeldanamycin in colon cancer cell lines results from inhibition of nuclear factor kappaB signaling. Cancer Res..

[36] Chocry M., Leloup L., Kovacic H. (2017). Reversion of resistance to oxaliplatin by inhibition of p38 MAPK in colorectal cancer cell lines: Involvement of the calpain / Nox1 pathway. Oncotarget.

[37] Saltz L.B. (1998). Irinotecan in the first-line treatment of colorectal cancer. Oncology (Williston Park).

[38] Pommier Y., Leo E., Zhang H., Marchand C. (2010). DNA topoisomerases and their poisoning by anticancer and antibacterial drugs. Chem. Biol..

[39] Rudolf E., Kralova V., Rudolf K., John S. (2013). The role of p38 in irinotecan-induced DNA damage and apoptosis of colon cancer cells. Mutat. Res..

[40] Tang W., Su G., Li J., Liao J., Chen S., Huang C., Liu F., Chen Q., Ye Y. (2014). Enhanced anti-colorectal cancer effects of carfilzomib combined with CPT-11 via downregulation of nuclear factor-κB in vitro and in vivo. Int. J. Oncol.

[41] Paillas S., Boissière F., Bibeau F., Denouel A., Mollevi C., Causse A., Denis V., Vezzio-Vié N., Marzi L., Cortijo C. (2011). Targeting the p38 MAPK pathway inhibits irinotecan resistance in colon adenocarcinoma. Cancer Res..

[42] Paillas S., Causse A., Marzi L., de Medina P., Poirot M., Denis V., Vezzio-Vie N., Espert L., Arzouk H., Coquelle A. (2012). MAPK14/p38α confers irinotecan resistance to TP53-defective cells by inducing survival autophagy. Autophagy.

[43] Chen M.C., Lee N.H., Hsu H.H., Ho T.J., Tu C.C., Hsieh D.J., Lin Y.M., Chen L.M., Kuo W.W., Huang C.Y. (2015). Thymoquinone induces caspase-independent, autophagic cell death in CPT-11-resistant lovo colon cancer via mitochondrial dysfunction and activation of JNK and p38. J. Agric. Food Chem..

[44] Tam S.Y., Wu V.W.C. (2019). A Review on the Special Radiotherapy Techniques of Colorectal Cancer. Front. Oncol..

[45] Cancer Stat Facts: Colorectal Cancer. https://seer.cancer.gov/statfacts/html/colorect.html.

[46] Fazeli M.S., Keramati M.R. (2015). Rectal cancer: A review. Med. J. Islam Repub. Iran.

[47] Dahlberg M., Glimelius B., Påhlman L. (1999). Improved survival and reduction in local failure rates after preoperative radiotherapy: Evidence for the generalizability of the results of Swedish Rectal Cancer Trial. Ann. Surg..

[48] Martinez-Useros J., Moreno I., Fernandez-Aceñero M.J., Rodriguez-Remirez M., Borrero-Palacios A., Cebrian A., Gomez Del Pulgar T., Del Puerto-Nevado L., Li W., Puime-Otin A. (2018). The potential predictive value of DEK expression for neoadjuvant chemoradiotherapy response in locally advanced rectal cancer. BMC Cancer.

[49] Wang Y., Li Y., Yang L., Yin D. (2017). Intermittent low dose irradiation enhances the effectiveness of radio- and chemo-therapy for human colorectal adenocarcinoma cell line HT-29. Oncol. Rep..

[50] De la Cruz-Morcillo M.A., García-Cano J., Arias-González L., García-Gil E., Artacho-Cordón F., Ríos-Arrabal S., Valero M.L., Cimas F.J., Serrano-Oviedo L., Villas M.V. (2013). Abrogation of the p38 MAPK α signaling pathway does not promote radioresistance but its activity is required for 5-Fluorouracil-associated radiosensitivity. Cancer Lett..

[51] Lafarga V., Cuadrado A., Lopez de Silanes I., Bengoechea R., Fernandez-Capetillo O., Nebreda A.R. (2009). p38 Mitogen-activated protein kinase- and HuR-dependent stabilization of p21(Cip1) mRNA mediates the G(1)/S checkpoint. Mol. Cell Biol..

[52] Zlobec I. (2013). Novel biomarkers for the prediction of metastasis in colorectal cancer. Expert Opin. Med. Diagn..

[53] Takeuchi K., Shin-ya T., Nishio K., Ito F. (2009). Mitogen-activated protein kinase phosphatase-1 modulated JNK activation is critical for apoptosis induced by inhibitor of epidermal growth factor receptor-tyrosine kinase. FEBS J..

[54] Montagut C., Iglesias M., Arumi M., Bellosillo B., Gallen M., Martinez-Fernandez A., Martinez-Aviles L., Cañadas I., Dalmases A., Moragon E. (2010). Mitogen-activated protein kinase phosphatase-1 (MKP-1) impairs the response to anti-epidermal growth factor receptor (EGFR) antibody cetuximab in metastatic colorectal cancer patients. Br. J. Cancer.

[55] Hou J., Lam F., Proud C., Wang S. (2012). Targeting Mnks for cancer therapy. Oncotarget.

[56] Wheater M.J., Johnson P.W., Blaydes J.P. (2010). The role of MNK proteins and eIF4E phosphorylation in breast cancer cell proliferation and survival. Cancer Biol. Ther..

[57] Furic L., Rong L., Larsson O., Koumakpayi I.H., Yoshida K., Brueschke A., Petroulakis E., Robichaud N., Pollak M., Gaboury L.A. (2010). eIF4E phosphorylation promotes tumorigenesis and is associated with prostate cancer progression. Proc. Natl. Acad. Sci. USA.

[58] Berger M.D., Stintzing S., Heinemann V., Yang D., Cao S., Sunakawa Y., Ning Y., Matsusaka S., Okazaki S., Miyamoto Y. (2017). Impact of genetic variations in the MAPK signaling pathway on outcome in metastatic colorectal cancer patients treated with first-line FOLFIRI and bevacizumab: Data from FIRE-3 and TRIBE trials. Ann. Oncol..

[59] Han Z., Zhu S., Han X., Wang Z., Wu S., Zheng R. (2015). Baicalein inhibits hepatocellular carcinoma cells through suppressing the expression of CD24. Int. Immunopharmacol..

[60] Chung H., Choi H.S., Seo E.K., Kang D.H., Oh E.S. (2015). Baicalin and baicalein inhibit transforming growth factor-β1-mediated epithelial-mesenchymal transition in human breast epithelial cells. Biochem. Biophys. Res. Commun..

[61] Choi E.O., Park C., Hwang H.J., Hong S.H., Kim G.Y., Cho E.J., Kim W.J., Choi Y.H. (2016). Baicalein induces apoptosis via ROS-dependent activation of caspases in human bladder cancer 5637 cells. Int. J. Oncol..

[62] Su M.Q., Zhou Y.R., Rao X., Yang H., Zhuang X.H., Ke X.J., Peng G.Y., Zhou C.L., Shen B.Y., Dou J. (2018). Baicalein induces the apoptosis of HCT116 human colon cancer cells via the upregulation of DEPP/Gadd45a and activation of MAPKs. Int. J. Oncol..

[63] Langenskiöld M., Holmdahl L., Angenete E., Falk P., Nordgren S., Ivarsson M.L. (2009). Differential prognostic impact of uPA and PAI-1 in colon and rectal cancer. Tumour. Biol..

[64] Wind T., Jensen J.K., Dupont D.M., Kulig P., Andreasen P.A. (2003). Mutational analysis of plasminogen activator inhibitor-1. Eur. J. Biochem..

[65] Jiang Y., Wu C., Boye A., Wu J., Wang J., Yang X., Yang Y. (2015). MAPK inhibitors modulate Smad2/3/4 complex cyto-nuclear translocation in myofibroblasts via Imp7/8 mediation. Mol. Cell Biochem..

[66] Wang X., Liu C., Wang J., Fan Y., Wang Z., Wang Y. (2017). Oxymatrine inhibits the migration of human colorectal carcinoma RKO cells via inhibition of PAI-1 and the TGF-β1/Smad signaling pathway. Oncol. Rep..

[67] Cheng J., Shah Y.M., Ma X., Pang X., Tanaka T., Kodama T., Krausz K.W., Gonzalez F.J. (2010). Therapeutic role of rifaximin in inflammatory bowel disease: Clinical implication of human pregnane X receptor activation. J. Pharmacol. Exp. Ther..

[68] Mencarelli A., Renga B., Palladino G., Claudio D., Ricci P., Distrutti E., Barbanti M., Baldelli F., Fiorucci S. (2011). Inhibition of NF-κB by a PXR-dependent pathway mediates counter-regulatory activities of rifaximin on innate immunity in intestinal epithelial cells. Eur. J. Pharmacol..

[69] Guzińska-Ustymowicz K., Pryczynicz A., Kemona A., Czyzewska J. (2009). Correlation between proliferation markers: PCNA, Ki-67, MCM-2 and antiapoptotic protein Bcl-2 in colorectal cancer. Anticancer Res..

[70] Esposito G., Gigli S., Seguella L., Nobile N., D’Alessandro A., Pesce M., Capoccia E., Steardo L., Cirillo C., Cuomo R. (2016). Rifaximin, a non-absorbable antibiotic, inhibits the release of pro-angiogenic mediators in colon cancer cells through a pregnane X receptor-dependent pathway. Int. J. Oncol..

[71] Mi C., Ma J., Wang K.S., Zuo H.X., Wang Z., Li M.Y., Piao L.X., Xu G.H., Li X., Quan Z.S. (2017). Imperatorin suppresses proliferation and angiogenesis of human colon cancer cell by targeting HIF-1α via the mTOR/p70S6K/4E-BP1 and MAPK pathways. J. Ethnopharmacol..

[72] Zhang Y., Wang X., Qin X., Liu F., White E., Zheng X.F. (2015). PP2AC Level Determines Differential Programming of p38-TSC-mTOR Signaling and Therapeutic Response to p38-Targeted Therapy in Colorectal Cancer. EBioMedicine.

